# Leaving the emergency department without complete care: disparities in American Indian children

**DOI:** 10.1186/s12913-018-3092-z

**Published:** 2018-04-10

**Authors:** Tess L. Weber, Katherine M. Ziegler, Anupam B. Kharbanda, Nathaniel R. Payne, Chad Birger, Susan E. Puumala

**Affiliations:** 1 Sanford Research, 2301 East 60th Street North, Sioux Falls, SD 57104 USA; 2 Colorado School of Public Health at the University of Colorado at Denver, 13001 E. 17th Place, B119, Bldg 500, 3rd Floor West Wing, Aurora, CO 80045 USA; 3 Children’s Hospitals and Clinics of Minnesota, 2525 Chicago Avenue South, Minneapolis, MN 55404 USA

**Keywords:** Pediatric, Emergency department, American Indian, Disparities

## Abstract

**Background:**

Children who leave the emergency department (ED) without complete evaluation or care (LWCET) have poorer outcomes in general. Previous studies have found that American Indian (AI) children have higher rates of LWCET than other racial or ethnic groups. Therefore, this study aims to examine LWCET in AI children by exploring differences by ED location and utilization patterns.

**Methods:**

This is a retrospective cohort study of five EDs in the upper Midwest between June 2011 and May 2012. We included all visits by children aged 0–17 who identified as African American (AA), AI or White. Logistic regression was used to determine differences in LWCET by race and ED location controlling for other possible confounding factors including sex, age, insurance type, triage level, distance from ED, timing of visit, and ED activity level.

**Results:**

LWCET occurred in 1.73% of 68,461 visits made by 47,228 children. The multivariate model revealed that AIs were more likely to LWCET compared to White children (Odds Ratio (OR) = 1.62, 95% Confidence Interval (CI) = 1.30–2.03). There was no significant difference in LWCET between AA and White children. Other factors significantly associated with LWCET included triage level, distance from the ED, timing of visit, and ED activity level.

**Conclusion:**

Our results show that AI children have higher rates of LWCET compared to White children; this association is different from other racial minority groups. There are likely complex factors affecting LWCET in AI children throughout the upper Midwest, which necessitates further exploration.

## Background

Leaving the emergency department (ED) without complete evaluation and treatment is a growing issue that can lead to serious medical conditions, additional cost, recurring ED/primary care visits, hospitalization or other preventable, adverse events [1–4]. Leaving without complete evaluation and treatment (LWCET) is frequently related to lack of timely treatment and length of stay before being seen, often resulting from overcrowded EDs [1–4]. Previous research has shown that racial and ethnic minorities have longer wait times than non-Hispanic White patients [2, 3, 5, 6]. This may be due, in part, to differential usage and care patterns in EDs, particularly in pediatric populations [5–8].

This is especially concerning among American Indian (AI) children, who have higher overall ED visit rates and more visits per child compared to other racial/ethnic groups [7]. Many factors contribute to this discrepancy, including a higher burden of chronic disease, substance abuse disorders, injuries (both intentional and unintentional), low insurance rates and lack of access to quality care [7–11]. Furthermore, many AI children lack a medical home, and therefore rely heavily on the ED for primary health care [8, 10].

Previous research has shown that AI children are more likely to LWCET [12]; however, no studies have thoroughly analyzed the patterns of use and care at the ED among AI children. This study aimed to examine LWCET in AI children by exploring possible differences by ED location and utilization patterns in a multi-center cohort of EDs in the upper Midwest.

## Methods

### Study design and sample

A cross-sectional study of pediatric ED visits from five EDs in the upper Midwest was performed. Included were all visits by children aged 0–17 years to one of the five EDs between June 2011 and May 2012. Analysis was limited to three racial groups (African American (AA), AI, and White). Approval from all Institutional Review Boards providing data was obtained, along with a waiver of informed consent.

### Statistical analysis

LWCET was the primary outcome measure, defined as leaving the ED without complete evaluation or treatment and recorded as a binary yes/no variable. Our primary exposure variable of interest was race, limited to AA, AI, and White since few other races were common at all of our study sites. Age was categorized as less than 1 year, 1–4 years, 5–10 years, and 11–17 years old. Insurance type was defined as private and medical assistance (MA)/other. A two-level triage variable was created where the three highest and two lowest triage levels were combined. High triage included emergent, critical and acute visits, while low triage included urgent and non-urgent visits. Sex was categorized as male and female. Timing of visit was developed based on day and time of visit to the ED. Categories included: weekday business hours, evening hours during a weekday, night hours during a weekday, and weekends. ED setting was categorized into rural or urban, where urban was an ED located in a city with a population of 100,000 people or more. Distance from the ED was based on the patient’s zip code, and categorized as 5 miles or less and more than 5 miles to the ED.

An important source of variation to consider in examining LWCET is how busy the ED is at the time the patient presents. For each site, we used partial least squares approach for structural equation models to construct a formative latent variable which measured the ED activity level. This variable takes into account the number of patients in each triage level at the emergency department as well as the time of day the patient arrived. The analysis was completed using the semPLS package in R with the centroid weighting scheme using R, version 3.2.3 (www.r-project.org). From this analysis, we created a standardized score for the activity level for each visit such that each site had a mean activity level near zero with positive number indicating increased activity and negative number indicating decreased activity. We checked basic validity of this construct by assessing its correlation with time to examination and found a Spearman’s rank correlation of 0.29 indicating a modest level of association.

Descriptive analysis including counts and percentages are reported. Univariate analyses were performed using logistic regression with only that variable and a random study site effect. Logistic regression with a random effect for study site was used to determine differences in LWCET by race and ED location controlling for other possible confounding factors including age, insurance type, triage level, sex, timing of visit, distance from the ED and ED activity level. Unadjusted and adjusted odds ratios are reported with 95% confidence intervals. Multiple imputations analysis was completed to examine the impact of missing data, which ranged from 0.04–1.08%. All logistic regression and imputations analyses were performed using SAS version 9.4, SAS Institute, Cary, NC.

## Results

### Demographics

During the 11-month study period, a total of 68,461 visits by 47,228 children were included in the analysis. Overall 1181 (1.73%) visits ended in LWCET (Table 1). The majority of visits were made by males (52.95%) and children between the ages of 1–4 (39.66%). Over half of the ED visits were made by White children (53.50%), while 36.66% and 9.84% were by AA and AI children, respectively. Most visits were triaged at less severe levels (57.10%) and were made by children with MA or other insurance (62.59%).

**Table 1:** Descriptive characteristics by leaving without complete evaluation and treatment (LWCET) status

Variable	Total (*N* = 68,461)	No LWCET (n, %)	LWCET (n, %)	*p*-value*
Sex				0.09
Female	32,207	31,680 (47.09)	527 (44.62)	
Male	36,254	35,600 (52.91)	654 (55.38)	
Race				< 0.001
AA	25,099	24,595 (36.55)	504 (42.68)	
White	36,624	36,094 (53.65)	530 (44.88)	
AI	6738	3591 (9.80)	147 (12.45)	
Age (years)				0.04
< 1	10,183	10,007 (14.87)	176 (14.90)	
1–4	27,152	26,646 (39.60)	506 (42.85)	
5–10	17,405	17,109 (25.43)	296 (25.06)	
11–17	13,721	13,518 (20.09)	203 (17.19)	
Insurance				0.09
Private	25,578	25,165 (37.42)	413 (35.00)	
MA/Other	42,853	42,086 (62.58)	767 (65.00)	
Triage Level				< 0.001
Less Severe	39,092	38,279 (57.50)	813 (70.70)	
More Severe	28,631	28,294 (42.50)	337 (29.30)	
Distance from ED (miles)				0.0014
≤ 5	34,043	33,401 (49.69)	642 (54.36)	
> 5	34,363	33,824 (50.31)	539 (45.64)	
Timing				< 0.001
Weekday Business Hours	18,233	17,931 (26.65)	302 (25.57)	
Weekday Evening Hours	17,772	17,412 (24.88)	360 (30.48)	
Weekday Night Hours	10,608	10,400 (15.46)	208 (17.61)	
Weekend	21,848	21,537 (32.01)	311 (26.33)	
24 Hour Repeat Visits				< 0.001
No Repeat Visits	67,308	66,192 (98.38)	1116 (94.50)	
Repeat Visit	1153	1088 (1.62)	65 (5.50)	
48 Hour Repeat Visits				< 0.01
No Repeat Visits	66,324	65,240 (96.97)	1084 (91.79)	
Repeat Visit	2137	2040 (3.03)	97 (8.21)	

LWCET indicates leaving without complete evaluation and treatment; *AA* African American, *AI* American Indian, *ED* Emergency Department

*P-values were produced by Chi-squared tests

The proportion of less severe triage scores was higher among children who LWCET compared to those who did not LWCET (70.70% vs. 57.50%), while percentages of more severe triage scores were lower among children who LWCET compared to those who did not LWCET (42.50% vs. 29.30%; Table 1). The proportion of return visits to the ED at ≤24 and ≤ 48 h was higher among LWCET visits (1.56% vs. 5.21% and 2.99% vs. 8.14% respectively, *P* < 0.001).

### Univariate analysis

AI children were more likely to LWCET compared to White children (Table 1). Children who LWCET were more likely to be triaged with less severe level (70.70%, *P* < 0.001), travel 5 miles or less to the ED (54.36%, *P* = 0.0014), be males (55.38%, *P* < 0.001), and visit during weekday evening hours (30.48%, *P* < 0.001) as compared to children who did not LWCET.

### Multivariate analysis

In the multivariate model there was a significant association between race and LWCET, where AI children had higher odds of LWCET than White children (OR = 1.62, 95% CI = 1.30–2.03; Table 2). There was no significant difference in LWCET between AA children and White children.

**Table 2:** Odds ratios of leaving without complete evaluation and treatment (LWCET)

Variable	Unadjusted	95% CI	Adjusted	95% CI	Imputed	95% CI
Sex						
Female	0.91	0.81–1.02	0.88	0.79–0.99	0.90	0.80–1.01
Race						
AA	1.40	1.23–1.58	1.06	0.90–1.24	1.05	0.89–1.23
AI	1.52	1.26–1.82	1.62	1.30–2.03	1.63	1.31–2.03
Age (years)						
< 1	0.93	0.78–1.10	0.88	0.73–1.05	0.91	0.76–1.08
5–10	0.91	0.79–1.05	0.89	0.77–1.04	0.91	0.78–1.05
11–17	0.79	0.67–0.93	0.89	0.75–1.05	0.90	0.76–1.06
Insurance						
Private	0.90	0.80–1.02	1.05	0.90–1.22	1.03	0.89–1.20
Triage Level						
More Severe	0.56	0.49–0.64	0.63	0.55–0.73	0.63	0.55–0.73
Distance from ED (miles)						
> 5	0.83	0.74–0.93	0.86	0.76–0.98	0.86	0.77–0.98
Timing						
Weekday Evening Hours	1.23	1.05–1.43	1.02	0.86–1.20	1.05	0.89–1.23
Weekday Night Hours	1.19	0.99–1.42	1.43	1.18–1.71	1.41	1.18–1.69
Weekend Hours	0.86	0.73–1.01	0.79	0.67–0.93	0.80	0.68–0.94
ED Activity Level						
	1.26	1.19–1.33	1.32	1.24–1.39	1.30	1.23–1.38

CI indicates confidence interval; *AA* African American, *AI* American Indian, *ED* Emergency Department

Triage level was significantly associated with LWCET, where children with more severe triage scores had lower odds of LWCET compared to those with less severe scores (OR = 0.63, CI = 0.55–0.73). Visits that occurred during weekday night hours had odds 1.43 times higher of LWCET compared to visits during weekday business hours (CI = 1.18–1.71). Children who lived further than 5 miles from the ED had lower odds of LWCET (OR = 0.86, CI = 0.76–0.98). ED activity level was also found to be associated with LWCET, where a busier ED had higher odds of resulting in LWCET (OR = 1.32, CI = 1.24–1.33).

### Imputations analysis

Results from imputations analysis revealed slight differences in associations with LWCET due to missing data values. For example, the odds of LWCET for children who lived further than five miles from the ED was found to be significantly lower compared to children who lived within five miles (OR = 0.86, CI = 0.77–0.98). In addition, the odds of LWCET for visits during weekend hours was significantly lower compared to visits during weekday business hours (OR = 0.80, CI = 0.68–0.94).

## Discussion

This study examined LWCET among pediatric patients in a multi-center cohort of EDs in the upper Midwest. Overall, 1.7% of pediatric visits resulted in LWCET. While these rates of LWCET are relatively low compared to other hospital settings, [1, 13, 14] results from this study found that AI children were significantly more likely to LWCET compared to white patients, which merits further discussion. Other factors associated with increased odds of LWCET included less severe triage score, presenting to the ED during weekday hours, and ED activity level.

Previous studies have found that patients who live closer to the ED have higher odds of leaving prematurely [1, 14]. This was also seen in our study, where children who lived further than 5 miles from the ED have slightly lower odds of LWCET (OR = 0.86, CI = 0.76–0.98). The assumption for this may be that patients who live closer to the ED may be more likely to use ED services than seeking other medical care because it may be quicker or more accessible than other options. Similarly, these patients may also leave the ED without being seen because they can return if necessary.

As location of the site could have been a factor in racial differences and LWBS, we tested the interaction between site and race and found no significant relationship. One may assume that the reason for differences in AI children may be because they are visiting rural EDs that may have fewer resources and longer wait times, resulting in a higher rate of LWCET. However, no difference in LWCET was seen between sites, and a higher amount of LWCET in AI children was seen regardless of ED location.

Both MA insurance and race may be proxies for low socioeconomic status, and patients with MA insurance often use the ED as a substitute for primary care [5, 12]. A previous study found that patients with low-income and poor, or no insurance, were more likely to leave without being seen [15]. However, no significant association was seen between LWCET and insurance in our study. This suggests that other factors, including social or cultural may be of importance when exploring the reasons for why AI children LWCET, and could explain why we observed a different association between AI children and LWCET than we observed with AA children.

Unique to our study, we examined the relationship of LWCET and the ED activity level. As previous research suggests [1–4], our results showed a significant association with higher ED activity levels and increased odds of premature departure. This may be associated with time of arrival, as EDs are undoubtedly busier at varying times. We also found a significant association with time of arrival and LWCET. Patients who presented to the ED on weekdays between 10 pm and 7 am had odds of LWCET 1.43 times higher than patients who arrived during weekday business hours. Contrary to previous research [1], however, our study found a lower rate of LWCET during the weekends.

The strongest predictor of LWCET was race. Previous studies have also found racial disparities among patient wait times and LWCET in the ED [2, 3, 5, 7, 16, 17]. We observed a significant relationship between AI children and LWCET while accounting for potential confounding factors. In our study, AI children had 1.62 times higher odds of LWCET compared to White children, whereas no significant difference was observed for AA children compared to White children. A similar study by Bourgeois et al. (2008) also found that AI children had higher odds of LWCET than White children [12]. This persistent increase in LWCET among AI children indicates significant disparities in ED care in this disadvantaged population, and merits further investigation.

Discrimination, or perceived discrimination, may play a role [18]. One study indicated that AI parents were 25 times more likely to perceive racial discrimination in a health care setting than non-Hispanic White patients [19]. Several studies have noted minority patients are often assigned less urgent triage scores, have longer wait times, and are less likely to receive pain medications in ED settings [20–22]. This may be due, in part, to health care providers’ conflicting attitudes towards patients who use the ED for non-urgent conditions [23]. Many social determinants may factor into these use patterns and lead to overuse and lower quality care. Understanding use and care patterns among AI pediatric patients in the ED will result in a more comprehensive picture of health care in this population. Interventions to address issues of ED use and care could lead to substantially improved ED outcomes for AI children.

### Strengths

This study had several strengths. The data encompasses five EDs with a larger proportion of AI children that allows us to look at patterns in LWCET in this specific minority group not currently explained within the literature. While limited by medical record data, we were still able to account for several confounders such as distance from ED, ED setting, timing of visit, and the level of traffic within the ED. The development of our ED activity level allows us to look more into the environment of the ED, while other studies have only focused on the day and time of visit without accounting for how busy the ED is at the time of visit.

### Limitations

There are potential limitations to this study. First, these data may not be representative of the AI children outside of the Upper Midwest since many regional differences may affect the relationship between race and LWCET; therefore, no assumptions should be made about generalizability outside this region. Furthermore, while we did attempt to account for differences by site by including it as a random effect in our model, the diversity of each site may affect the results and the EDs included may not be a random sample of the population of EDs that serve AI children. Finally, medical record data did not allow us to determine at what point in the visit children LWCET or for what reason, therefore we are only able to suggest further exploration into this observed relationship to better inform possible intervention efforts.

## Conclusions

Results of this study show a significant relationship between LWCET and race. Specifically, AI children have a significantly higher rate of LWCET compared to White children regardless of ED location. This was not seen among AAs. This implies that underlying factors attribute to AI children LWCET throughout the upper Midwest.

## Abbreviations

AA: African American
AI: American Indian
CI: 95% Confidence interval
ED: Emergency department
LWCET: Leaving without complete evaluation and treatment
MA: Medical assistance
OR: Odds ratio
